# Novel use of riociguat in infants with severe pulmonary arterial hypertension unable to wean from inhaled nitric oxide

**DOI:** 10.3389/fped.2022.1014922

**Published:** 2022-12-01

**Authors:** L. T. Domingo, D. D. Ivy, S. H. Abman, A. M. Grenolds, J. T. MacLean, J. A. Breaux, K. J. Minford, B. S. Frank

**Affiliations:** ^1^ Department of Pediatrics, Primary Children's Hospital, University of Utah, Salt Lake, UT, United States; ^2^ Department of Pediatrics, Children's Hospital of Colorado, University of Colorado, Aurora, CO, United States

**Keywords:** riociguat, pediatric pulmonary arterial hypertension, pulmonary vasodilator, targeted therapy, pulmonary vasoreactivity, TBX4 mutation, SLC25A26 mutation

## Abstract

**Introduction:**

Riociguat, an oral soluble guanylate cyclase stimulator, has been approved for use in adults with pulmonary arterial hypertension (PAH) and chronic thromboembolic pulmonary hypertension. However, there is limited data on its therapeutic use in children.

**Case Presentation:**

We report the case of two infants with severe suprasystemic pulmonary hypertension who were successfully treated with riociguat after failure to wean off inhaled nitric oxide (iNO) despite combination PAH therapy. Case 1 is a 6-month-old term male with TBX4 deletion who presented with severe hypoxemic respiratory failure and severe PAH immediately after birth. Initial cardiac catheterization showed PVRi 15.5 WU*m2. Marked hypoxemia and PAH persisted despite aggressive therapy with sildenafil, bosentan, intravenous treprostinil, and milrinone. The infant required high doses of inhaled nitric oxide (60 ppm) and manifested significant post-ductal hypoxemia and hemodynamic instability with any attempt at weaning. After discontinuation of sildenafil, initiation, and very slow uptitration of riociguat, the patient was able to maintain hemodynamic stability and wean from nitric oxide over 6 weeks with persistently severe but not worsened pulmonary hypertension. Case 2 is a 4-month-old term male with compound heterozygous SLC25A26 mutation and severe pulmonary hypertension. Initial cardiac catheterization showed PVRi 28.2 WU*m2. After uptitration of sildenafil, bosentan, and IV treprostinil, serial echocardiograms continued to demonstrate near-systemic pulmonary hypertension. He failed multiple attempts to wean off typical doses of iNO (10–20 ppm) over the following weeks with tachypnea, hypoxemia, and worsening pulmonary hypertension on echocardiogram despite continued aggressive combination targeted therapy. After a 24-h sildenafil washout, he was initiated and uptitrated on riociguat with concomitant, successful wean of nitric oxide over one week that was well tolerated. No serious adverse effects in the titration period were observed.

**Conclusion:**

Riociguat may be considered as an adjuvant therapeutic agent in selected children with severe PAH who are poorly responsive to sildenafil therapy and unable to wean from iNO.

## Introduction

Pulmonary arterial hypertension (PAH) is associated with poor prognosis and without treatment, can lead to right heart failure and death (1). In recent years, prognosis has improved with the introduction of new PAH - targeted therapies (2, 3). Current approved PAH therapies focus on pulmonary vasodilation and target three different pathways: prostacyclin, endothelin, and nitric oxide (NO)-soluble guanylate cyclase (sGC)-cyclic guanosine monophosphate (cGMP) pathways (4, 5). However, management of pediatric pulmonary hypertension (PH) remains challenging due in part to the shortage of pediatric-specific clinical trials. Treatment of children is largely based on clinical experience and adult data, and the pediatric use of most PAH targeted therapies is off-label (5).

Riociguat is a drug that acts on the NO-sGC-cGMP pathway which has been approved for use in adults with PAH and chronic thromboembolic pulmonary hypertension (6–8). Unlike phosphodiesterase-5 (PDE5) inhibitors that block the degradation of cGMP, riociguat acts as a direct stimulator of sGC to increase cGMP production in a manner similar to the mechanism for inhaled nitric oxide (iNO). Recent literature supports the efficacy and tolerability of riociguat as an alternative agent for adults with PAH who have insufficient response to treatment with PDE5 inhibitors (7, 9–13). The use of riociguat in pediatric PAH, however, has not been approved because of limited data on its therapeutic use in children (14).

We report the case of two infants with severe suprasystemic PAH who failed to wean from iNO despite combination PH-targeted drug therapy that included sildenafil but were successfully weaned from iNO with the addition of riociguat therapy.

## Case reports

**Figure 1:**
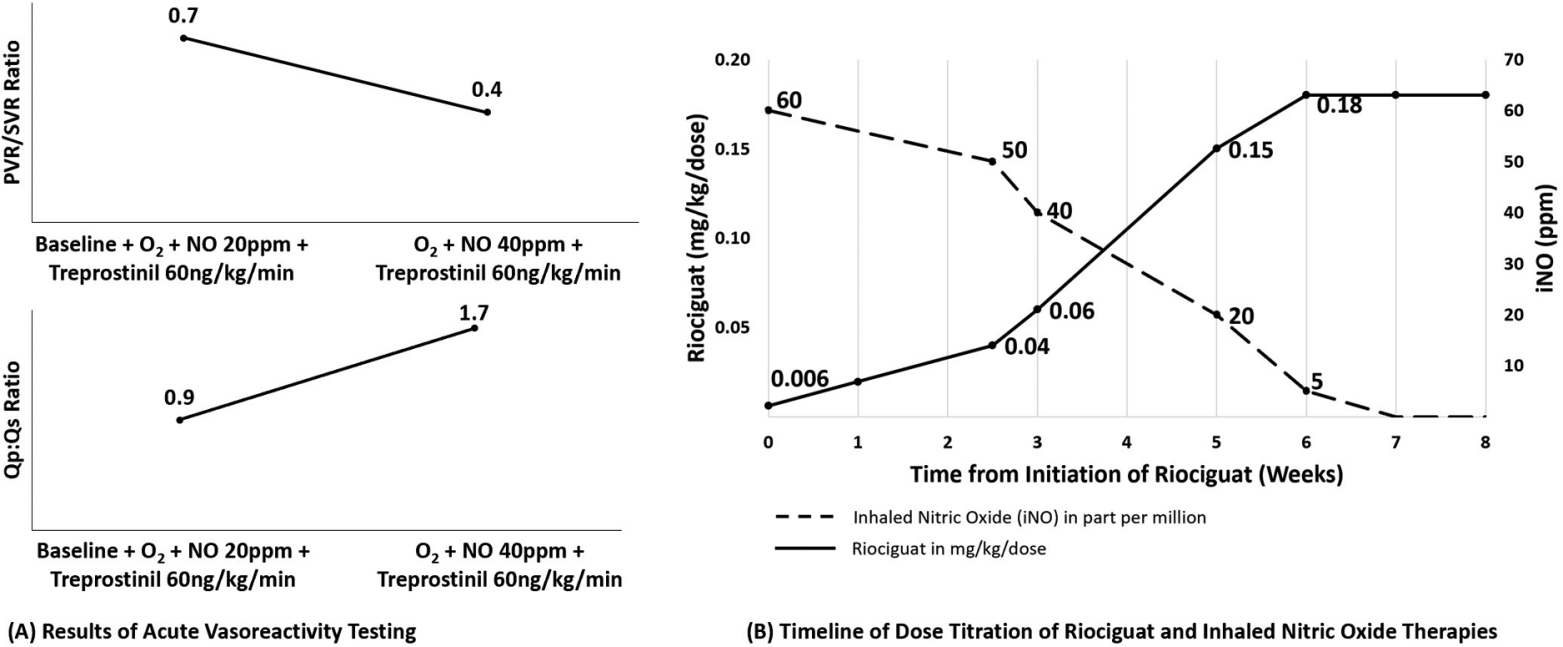
Results of acute vasoreactivity testing and timeline of dose titration of riociguat and inhaled nitric oxide therapies for patiennt 1. (**A**) Results of hemodynamic evaluation for patient 1. With acute increase in nitric oxide dose delivery, we observed (**A**) decreased pulmonary vascular resistance and (**B**) increased pulmonary blood flow despite background therapy with sildenafil, treprostinil, high fractional inspired oxygen, and nitric oxide at 20 ppm. (**B**) Timeline for initiation of riociguat therapy and weaning off inhaled nitric oxide. Riociguat therapy was started at very low dose (0.006 mg/kg/dose = 0.05 mg total dose once per day) and uptitrated slowly over 7 weeks to a dose of 2 mg/dose TID while nitric oxide was weaned over a similar timeframe. Not shown, over the subsequent 6 months his dose was titrated up to final dose of 2.5 mg TID. O_2_, oxygen; NO, inhaled nitric oxide; PVR, pulmonary vascular resistance; SVR, systemic vascular resistance; Qp, pulmonary flow; Qs, systemic flow.

Patient 1 is a 6-month-old boy who was born term with a birth weight of 3.3 kg *via* urgent C-section due to maternal bleeding. Prenatal course was otherwise unremarkable. Due to severe hypoxemic respiratory failure at birth, he was treated with intubation, high frequency ventilation, supplemental oxygen (FiO2 = 1.00), iNO, and surfactant administration. Echocardiogram showed right to left ductal shunt consistent with severe PH. Intravenous epoprostenol, sildenafil and milrinone were started in addition to iNO. He underwent cardiac catheterization at 1.5 months of age. Hemodynamic evaluation showed an indexed pulmonary vascular resistance (PVRi) of 15.5 WU*m2 (iNO, oxygen and sildenafil) which decreased to 10.2 WU*m2 with acute uptitration of epoprostenol. Chest CT scan was significant for enlarged pulmonary arteries, patent ductus arteriosus and nonspecific findings suggestive of interstitial lung disease including interlobular septal thickening and ground-glass opacity without pulmonary nodules. He subsequently underwent lung biopsy which demonstrated alveolar simplification with interstitial widening, muscularization of small and medium sized pulmonary arteries and interstitial cells suggestive of pulmonary interstitial glycogenosis but no evidence of alveolar capillary dysplasia. Whole exome sequencing showed a pathogenic 17q23 microdeletion, which encompasses the PH associated TBX4 gene. He was then transferred to our institution at ∼2 months of age for further care. His PAH therapy was titrated to combination therapy with sildenafil, bosentan, intravenous treprostinil, and iNO. His treatment also included an intravenous steroid burst followed by daily inhaled steroids with a modest clinical response but persistent severe pulmonary hypertension by echocardiogram. Over the subsequent 2 months, he continued to manifest hemodynamic instability and post-ductal hypoxemia suggestive of suprasystemic PH. At 4 months of age sildenafil was discontinued due to the lack of a clear clinical response as well as parental concerns regarding the use of this drug. He also developed significant transaminitis prompting cessation of bosentan at 5.5 months of age. He subsequently underwent a second cardiac catheterization at 6 months of age to evaluate his response to ongoing therapy, which showed improvement from his previous catheterization but still severe disease, with a mean pulmonary arterial pressure (mPAP) of 38 mmHg [systemic mean arterial pressure (sMAP) of 49 mmHg], and PVRi of 5.91 WU*m2 (iNO + oxygen + treprostinil). His Qp:Qs improved with increasing doses of iNO and treprostinil (Figure 1A). Due to hypotension after the catheterization, his treprostinil dose was maintained at 50 ng/kg/min and iNO increased to 60 ppm delivered *via* noninvasive ventilation. He failed multiple attempts to wean his iNO dose below 60 ppm, as evidenced by right to left ductal shunting on echocardiogram and significant pre- and post-ductal saturation gradients during attempted weans. Because of inability to even slowly wean from this high dose of iNO, the decision was made to start riociguat as additional targeted PAH therapy. Riociguat was initiated at 0.05 mg once daily (0.006 mg/kg/dose), and slowly increased to 0.5 mg/dose thrice daily. He had hypotension without signs of inadequate perfusion after the third dose of riociguat but responded well to saline bolus. Apart from this event, the titration was well tolerated and his hypoxemic events became less frequent. Riociguat was gradually increased to 2 mg/dose TID with iNO weaned and eventually discontinued 7 weeks after riociguat initiation (Figure 1B). He had no other reported side effects from riociguat during the titration period. During riociguat uptitration, he was able to wean from noninvasive positive pressure ventilation to nasal cannula. Repeat echocardiogram off iNO showed improved ventricular septal flattening and a greater degree of left to right shunting across the PDA, suggesting improved pulmonary vascular resistance. He was discharged home on subcutaneous treprostinil 37 ng/kg/min, riociguat 2 mg TID, and supplemental oxygen. Repeat echocardiogram 2 months later showed continuous low velocity left to right flow in his patent ductus arteriosus, moderate septal flattening and preserved biventricular systolic function. With improved growth and activity level, and no parental report of intolerance to medications after 4 months on this combination therapy, riociguat was increased to 2.5 mg TID at 13 months of age (body weight 9.4 kg). At latest outpatient follow up, he was 21 months old, World Health Organization functional class II with persistent sub-systemic pulmonary hypertension by echocardiogram, and preserved growth.

**Figure 2:**
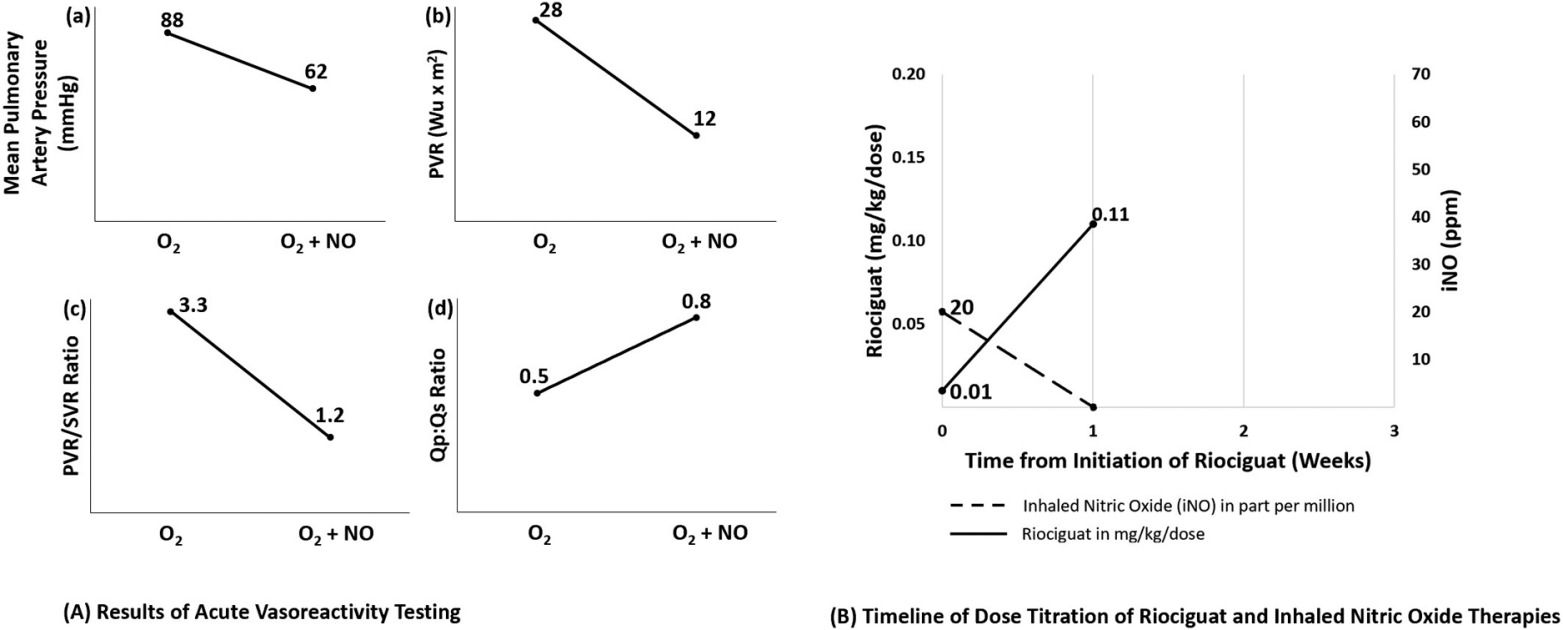
Results of acute vasoreactivity testing and timeline of dose titration of riociguat and inhaled nitric oxide therapies for patient 2. (**A**) Results of acute vasoreactivity testing for patient 2. Acute challenge with increased oxygen (FiO2 1.0) and addition of inhaled nitric oxide at 40 ppm resulted in significant improvement of (**A**) mean pulmonary artery pressure, (**B**) indexed pulmonary vascular resistance, (**C**) PVR:SVR ratio and (**D**) Qp:Qs ratio. (**B**) Timeline for initiation of riociguat therapy and weaning off inhaled nitric oxide. Patient 2 was started on riociguat 0.01 mg/kg/dose (0.05 mg total dose three times per day) with a daily dose increase, achieving 0.5 mg/dose TID after 1 week. Nitric oxide was weaned over that same week to off without adverse clinical sequelae noted. Not shown, his dose was titrated up to 2 mg TID over the subsequent four months. O_2_, oxygen; NO, inhaled nitric oxide; PVRi, indexed pulmonary vascular resistance; PVR, pulmonary vascular resistance; SVR, systemic vascular resistance; Qp, pulmonary flow; Qs, systemic flow.

Patient 2 is a 4-month-old male born at 38 weeks gestational age. Family history was significant for an 18-month-old sibling with SLC25A26 mutation and severe PAH. Given the family history, he was admitted to the neonatal intensive care unit for observation and was found to have the same compound heterozygous SLC25A26 mutation and severe PAH. He was discharged home during the first week of life with no complications. At 3 weeks of age, he developed tachypnea and poor feeding. Echocardiogram showed moderate septal flattening, moderate right heart dilation and preserved biventricular systolic function. Sildenafil was initiated and he was transferred to our institution. Initial echocardiogram at our institution showed suprasystemic RV systolic pressures and moderate RV dysfunction despite sildenafil therapy. Cardiac catheterization at 7 weeks of age showed mPAP 88 mmHg (sMAP 56 mmHg) and PVRi 28.2 WU*m2 (oxygen), which decreased to mPAP 62 mmHg (sMAP 59 mmHg), and PVRi 12 WU*m2 (iNO + oxygen) (Figure 2A). Bosentan, intravenous treprostinil and iNO were added. Despite escalation of PAH therapy with uptitration of intravenous treprostinil to 65 ng/kg/min, he continued to show systemic PH. He also received multiple intravenous steroid bursts with improvement in work of breathing and oxygen requirement but only modest improvement in PAH severity by echocardiogram. He failed multiple attempts to wean off iNO *via* noninvasive ventilation over the following weeks with tachypnea, hypoxemia, and worsening PH on echocardiogram. Due to insufficient response to his combination therapy and inability to wean from iNO, he was started on riociguat at a dose of 0.05 mg TID (0.01 mg/kg/dose) after sildenafil had been held for 48 h. We advanced the riociguat by 0.1 mg/dose daily to initial goal of 0.5 mg/dose TID without evidence of adverse effects. He tolerated a slow wean off iNO and noninvasive ventilation to oxygen supplementation *via* nasal cannula without clinical complications and with improved PAH by echocardiogram over the course of 1 week (Figure 2B). Over the following months, riociguat was gradually uptitrated in 0.1 mg/dose intervals to eventual target dose of 2 mg/dose TID. He was able to be discharged home 2 weeks later on riociguat, bosentan, and subcutaneous treprostinil. At 5 months of age, he was re-admitted with Covid-19 pneumonia and had a prolonged hospitalization that included worsening of his PH, uptitration of treprostinil and re-initiation of iNO. At latest follow up, he is 11 months old and managed as an outpatient on supplemental oxygen, riociguat, bosentan, and treprostinil.

## Discussion

**Figure 3:**
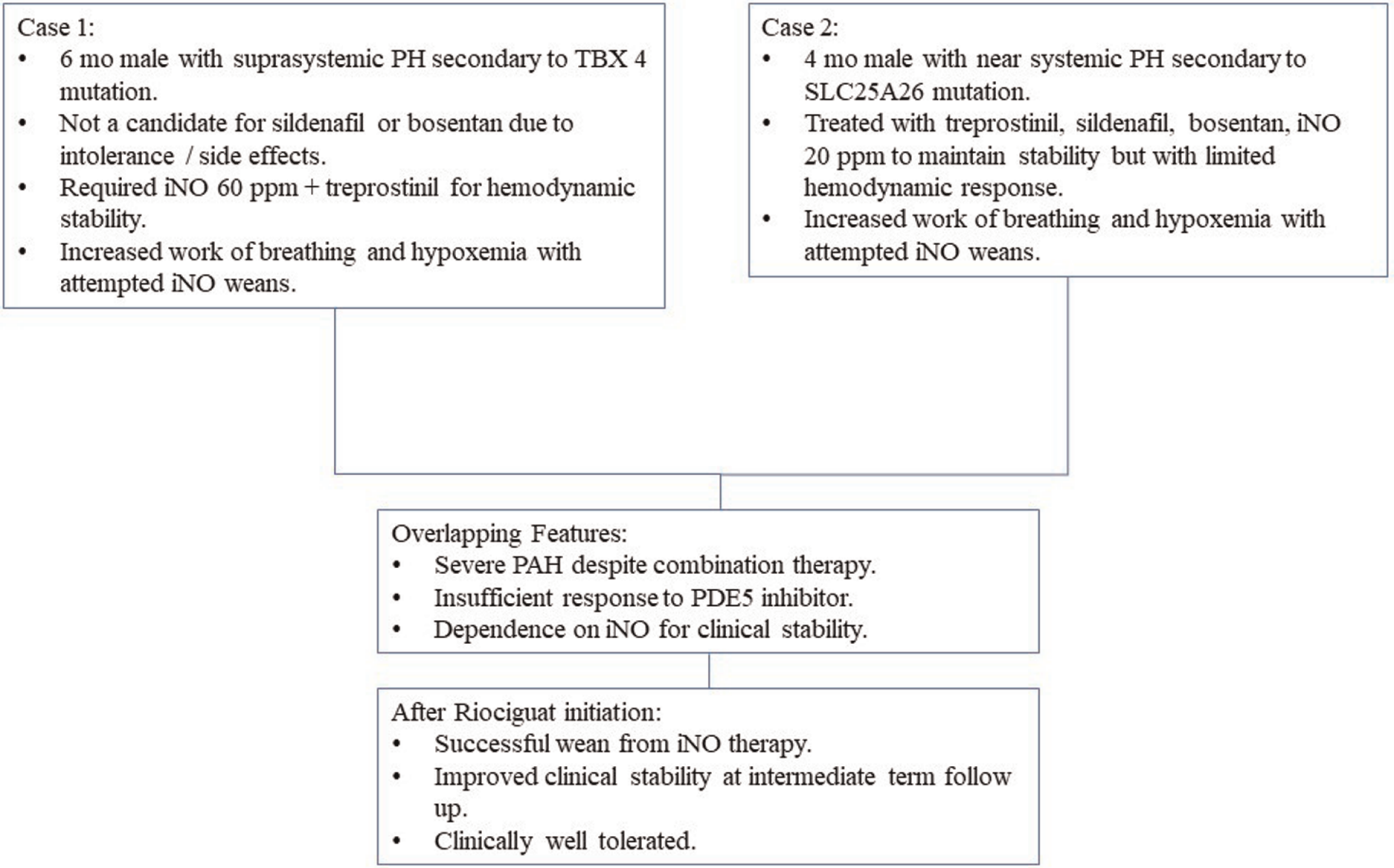
Summary of clinical cases.

We report successful transition from sildenafil and iNO to riociguat therapy in two infants with severe PAH who were poorly responsive to sildenafil therapy and had demonstrated difficulty in tolerating even modest weans in iNO therapy (Figure 3). To the best of our knowledge, our patients are the youngest in the reported literature to be treated with riociguat. These cases suggest that riociguat merits further investigation for the subset of patients with severe PH who demonstrate need for very high dose iNO to achieve stability or those with clinical sensitivity to withdrawal of iNO, especially in the setting of poor responsiveness to sildenafil therapy.

Given the considerable hemodynamic response to iNO both clinically and during cardiac catheterization, and the clinically observed sensitivity to iNO weans, both infants were started on riociguat with initial dose of 0.05 mg/dose as extrapolated from the case report of the successful treatment of a child with riociguat (14). In both cases, riociguat was prescribed using the clinical judgement of the treating team, not within the context of a clinical trial. Recognizing the limited data and uncertain risk of riociguat use in children, we utilized shared decision making in choosing to initiate and titrate the riociguat. It is important to note that riociguat must not be used in combination with PDE5 inhibitors because of the risk of severe systemic arterial hypotension (15). In addition, riociguat should be avoided in those with hepatic or renal impairment (16, 17). In both our patients, sildenafil was discontinued for at least 48 h before riociguat therapy was initiated. Patient 1 had transaminitis (ALT 3.4x elevated, AST 1.2x elevated) but normal synthetic liver function. His transaminitis improved ∼6 months after discontinuing bosentan. Both our patients had normal renal function. No clinical worsening occurred during the sildenafil-free period. Both tolerated slow riociguat advance with iNO gradually weaned to prevent rebound pulmonary hypertension (18–21).

Our approach to these patients is supported by the experience in adult PAH. Recent literature reports clinical efficacy of transitioning PDE5 inhibitors to riociguat for those who do not reach treatment goals. The pivotal study PATENT-1 is a double-blind randomized placebo-controlled trial of PAH patients. In this study, patients who were treated with riociguat had improvement in 6-minute walk distance (6MWD), pulmonary vascular resistance (PVR), N-terminal prohormone of brain natriuretic peptide (NT-proBNP), World Health Organization functional class (WHO-FC), time to clinical worsening, and Borg dyspnea scale rating (7). These improvements were sustained in the long term extension study PATENT-2 (12, 13). Similar findings were observed in the RESPITE study (9). RESPITE is an open label study that investigated the benefits of switching from stable doses of sildenafil or tadalafil to riociguat in PAH patients who remain symptomatic and have insufficient response to PDE5 inhibitors. At the end of the study period, improvements were seen in the 6MWD, NT-proBNP, WHO-FC and hemodynamic parameters. However, despite these studies that report improved treatment efficacy after switching from PDE5 inhibitors to riociguat in adults, there is very limited data or experience with use of riociguat in children (22–24).

The clinically observed greater efficacy with riociguat compared to sildenafil in our patients may be explained by its dual mode of action on sGC. Since riociguat works by both increasing the sensitivity of sGC to endogenous NO by stabilizing NO-sGC binding, and directly stimulating sGC independent of NO (25), the effect of riociguat may be greater in certain PAH patients who have a defective NO-sGC-cGMP pathway leading to insufficient or non-sustained response to PDE5 inhibitors (26–33). In addition, it has been shown that other phosphodiesterases could degrade cGMP in the presence of PDE5 inhibition, causing PDE5 inhibitors to be less effective (34).

Several preclinical studies provide evidence of mechanisms by which riociguat could be preferable to sildenafil in certain PAH populations. The efficacy of riociguat and sildenafil in the setting of hypoxia, was compared in studies involving rat and human pulmonary arteries (35). While both riociguat and sildenafil inhibited hypoxic pulmonary vasoconstriction, riociguat was found to be more effective as vasodilator than sildenafil with near maximal relaxation compared to ∼50% relaxation with sildenafil. Moreover, riociguat was 3-fold more potent under hypoxic conditions and did not worsen ventilation-perfusion coupling. The authors hypothesized that the observed difference was due to low basal NO activity leading to insufficient cGMP despite strong PDE5 inhibition. Alternatively, riociguat may also be more effective under chronic states of hyperoxia. Studies have shown that exposure of neonatal lungs to supraphysiologic oxygen levels may induce cellular dysfunction that can persist beyond the neonatal period (35–39), and even brief exposure to hyperoxia can lead to inactivation of endothelial NO synthase (eNOS), decreased sGC responsiveness to NO, increased PDE5 activity, and induction of mitochondrial reactive oxygen species which directly increase PDE5 activity (33, 35–37). In addition, exposure of neonatal lungs to oxidative stress affects the NO-sGC-cGMP pathway through oxidation of the heme-bound sGC (39–42). The decreased responsiveness of sGC to NO in such states may lead to lower levels of cGMP. In this scenario, riociguat could be superior to sildenafil by directly stimulating sGC, thereby increasing cGMP generation rather than decreasing breakdown through PDE5 inhibition (39). Similarly, increased cGMP levels after oxidative stress and greater pulmonary vasodilation compared to oxygen, iNO, acetylcholine or sildenafil were observed in earlier studies of the sGC activator cinaciguat in ovine models of pulmonary hypertension (41–43). These provide more evidence of the effectiveness of sGC modulators in conditions of oxidative stress leading to increased concentrations of NO-insensitive sGC and low cGMP levels.

In addition to the vasodilatory effects of riociguat noted in these studies, several pre-clinical studies have also shown antiproliferative, antifibrotic, and anti-inflammatory effects of riociguat and other sGC stimulators (26, 44–49) such that riociguat may have beneficial effects on multiple pathologic mechanisms contributing to PH. A study involving neonatal rats suggested that riociguat may prevent hyperoxia-induced lung injury with decrease in lung inflammation, improvement of both lung alveolar and vascular development, and decrease in vascular remodeling (46). Stimulation of soluble guanylate cyclase also reversed RV hypertrophy (RVH) and pulmonary vascular remodeling in mice models (26, 45). In another study using bleomycin-exposed mice, riociguat, compared to sildenafil, significantly improved pulmonary fibrosis and pulmonary inflammation in addition to its effect on PH and RVH (47). Similarly, riociguat significantly decreased RVH, increased cardiac output, and decreased total PVR compared with sildenafil in rats with severe PAH induced by hypoxia and the vascular endothelial growth factor receptor antagonist SU5416 (48). Compared with sildenafil, the effects of riociguat on RV function, and the neointima/media ratio of pulmonary arteries were significantly better. Furthermore, riociguat was shown to prevent the development of PH, RVH and vascular remodeling, as well as reduce inflammatory cell infiltrate and apoptosis of alveolar and endothelial cells in the lungs compared with controls in mouse model of chronic obstructive pulmonary disease and PH (49). These secondary effects may lead to improvement of cardiac hemodynamics and lung function, and therefore, pulmonary hypertension. There is also increasing evidence that maladaptation of the inflammatory and immune systems contribute to pulmonary vascular remodeling and PH such that therapies that directly modulate inflammatory processes have become a recent focus of clinical studies in PAH (50, 51). Both our patients received systemic and inhaled steroids at various times with improvement in work of breathing and oxygen requirement but only modest improvement in PAH severity. From this mixed clinical observation, it is difficult to say what, if any, role pulmonary inflammation played in each patient's clinical course, but this is an important area for future study generally in PAH and specifically in the utility of riociguat.

Lastly, riociguat may have the additional benefit of eliminating the potential direct interaction between bosentan and sildenafil (52), thus improving the therapeutic plasma concentrations and efficacy of both medications.

Overall, riociguat has been well tolerated in adult clinical studies. The most commonly reported drug-related adverse events in ∼40–50% of the patients include hypotension, syncope, dyspepsia, nausea, vomiting, dizziness, headache, cough and upper respiratory infections (7, 9, 10, 12, 13). Adverse events leading to discontinuation of riociguat were reported in 3%–9% of patients (7, 9, 10, 53). Although most of the adverse events were not considered serious, there were cases of significant hemoptysis and pulmonary hemorrhage seen in these studies (7, 9, 12). In our experience, Patient 1 had borderline hypotension without signs of inadequate end organ perfusion during the initiation period of riociguat that resolved after a single 10 cc/kg saline bolus. No other adverse effects in the titration period or follow up were observed in either case, which is similar to the report by Spreeman (14). Although our patients are too young to describe symptoms, there was no increase in fussiness, changes in activity or worsening feeding intolerance noted that may indicate these reported drug-related adverse events. Additionally, a previous study demonstrated dose-related adverse effects of sGC agonists on long bone growth including bone resorption and variable bone formation (54). Due to the reported multifocal bone changes in rats given riociguat, Patient 1 had serial hand radiographs which did not reveal any bone changes. This is consistent with the study in neonatal rats treated with riociguat which showed no effects on bone growth or structure (46). PATENT-CHILD, an open label study designed to evaluate the safety, tolerability, pharmacodynamics, and pharmacokinetics of riociguat in children with PAH aged 6–17 years old, is currently ongoing and may provide additional support on the use of riociguat in pediatric PAH (NCT02562235) (55).

Our study has several limitations. First, this is an observational study of only two patients with intermediate duration of follow up and so we are unable to evaluate efficacy and safety of long-term therapy with riociguat. We also cannot identify characteristics or risk factors of patients who may have a more favorable response to early treatment with riociguat instead of a PDE5 inhibitor. Since both our patients have heritable PAH, the pattern of their genetic variants may have influenced their response to PDE5 inhibitor and riociguat therapies. Future precision-medicine studies of how patients with different subtypes of group 1 PAH respond to different targeted therapies will be important (56, 57). Our reported outcomes are also limited to clinical and echocardiographic data without repeat hemodynamic data. Since this is performed at an altitude of 1,600 meters, the hemodynamic measurements and the observed response of our patients to both riociguat and PDE5 inhibitor may also vary at different elevations. Additionally, our approach to initial dosing and titration evolved based on our previous experience and individual patient response, and may not be applicable to the entire pediatric population. An in-depth discussion of the mechanisms of action and dosing of all PAH therapies used in these patients is beyond the scope of our report. Finally, although we did not see any adverse events after stopping sildenafil prior to initiating riociguat, interruption of this therapy remains a major concern especially in critically-ill patients. Future studies are necessary to determine the optimal dosing, drug-free and dose adjustment periods for riociguat in children.

## Conclusion

Our report suggests that riociguat may be considered as an adjuvant therapeutic agent in selected children with severe PAH who are poorly responsive to sildenafil and unable to wean from iNO therapy. Further studies are needed to better define criteria for determining the efficacy and safety of riociguat use in young children, and to determine the subset of patients who will benefit from early therapy with riociguat instead of PDE5 inhibitors.

## Data Availability

The raw data supporting the conclusions of this article will be made available by the authors, without undue reservation.
